# Dysfunction in Cardiovascular Autonomic Modulation Caused by Chronic Use of Ketamine Hydrochloride Can Be Prevented by Aerobic Exercise Training in Wistar Rats

**DOI:** 10.3390/jcm14217548

**Published:** 2025-10-24

**Authors:** Adriano Dos-Santos, Lucas Porto Fernandes dos Santos, Gabriela da Silva-Santos, Bruno Durante da Silva, Bruno Nascimento-Carvalho, Hunter Douglas de Souza-Lima, Nicolas da Costa-Santos, Erico Chagas Caperuto, Nathalia Bernardes, Katia De Angelis, Maria Claudia Irigoyen, Katia Bilhar Scapini, Iris Callado Sanches

**Affiliations:** 1 Human Movement Lab, Sao Judas Tadeu University, Sao Paulo 03166-000, Brazil; 2 Heart Institute, University of São Paulo, Sao Paulo 05508-220, Brazil; 3 Department of Physiology, Federal University of São Paulo, Sao Paulo 04021-001, Brazil

**Keywords:** ketamine, aerobic exercise, cardiovascular autonomic control

## Abstract

**Background/Objectives:** Ketamine, widely used for its anesthetic and analgesic properties, has been linked to cardiotoxic effects, particularly with chronic use. Prolonged ketamine exposure may impair cardiovascular function, while aerobic exercise is known to promote protective cardiovascular adaptations. This study aimed to evaluate whether aerobic training can mitigate the deleterious cardiovascular effects of chronic ketamine administration in rats. **Methods:** Twenty-four Wistar rats were randomly assigned to four groups: sedentary control (S), trained control (T), sedentary with ketamine (S-ket), and trained with ketamine (T-ket). Ketamine was administered intraperitoneally at a dose of 10 mg/kg, three times per week for six weeks. Aerobic training was conducted on a treadmill in the trained groups throughout the protocol. At the end of the experiment, cardiac function was assessed by echocardiography. Additionally, animals were cannulated in the carotid artery and jugular vein to measure blood pressure, baroreflex sensitivity, and heart rate variability using a data acquisition system (2 kHz, Windaq DATAQ). **Results:** Rats in the S-ket group showed elevated systolic arterial pressure and reduced baroreflex sensitivity compared to controls. Aerobic training attenuated these effects. Baroreflex sensitivity improved (bradycardic reflex—S: −1.7 ± 0.3; S-ket: −0.7 ± 0.1; T: −1.3 ± 0.2; T-ket: −1.3 ± 0.1), and cardiovascular autonomic function was preserved (total power—S: 45.6 ± 6.3; S-ket: 18.3 ± 2.1; T: 44.1 ± 5.7; T-ket: 38.9 ± 8.4) in trained animals. **Conclusions:** Aerobic exercise mitigates cardiovascular impairments caused by chronic ketamine exposure in rats, suggesting its potential as a non-pharmacological intervention to counteract ketamine-induced cardiotoxicity. These findings support incorporating exercise into treatment strategies for individuals chronically exposed to ketamine.

## 1. Introduction

Ketamine, initially developed as an anesthetic and analgesic, displays a wide range of pharmacological properties, including dissociative, hallucinogenic, and stimulatory effects [1,2]. Designed as a safer alternative to phencyclidine, ketamine has become a valuable tool in medical practice due to its unique pharmacodynamic profile.

Since the early 2000s, ketamine has gained increasing attention for its rapid antidepressant effects, particularly in cases of treatment-resistant depression, where conventional antidepressants fail to produce adequate responses in up to 30% of patients [3,4,5]. Unlike traditional therapies, which may take weeks to exert clinical effects, ketamine can alleviate depressive symptoms within hours [6]. In addition to psychiatry, ketamine has shown efficacy in pain management, especially for chronic and neuropathic pain conditions such as complex regional pain syndrome type I [7,8,9].

However, despite its therapeutic promise, ketamine use—especially when prolonged—raises concerns regarding cardiovascular safety. Common side effects include increased blood pressure and heart rate, palpitations, arrhythmias, and hemodynamic instability [10,11]. Evidence suggests that these effects may be mediated by oxidative stress in cardiac tissue, with chronic exposure exacerbating such outcomes and increasing the risk of cardiotoxicity [12].

The long-term cardiovascular effects of ketamine remain poorly understood, particularly regarding autonomic regulation. Some studies report heightened sympathetic activity, electrical disturbances, and cardiomyocyte apoptosis following repeated exposure [13]. Although pharmacological agents have been proposed to mitigate these effects, alternative strategies such as aerobic exercise may offer safer, non-pharmacological protection. In this context, a recent systematic review by Gullett et al. [14] emphasized the role of parasympathetic regulation—measured via heart rate variability (HRV)—as a marker of emotional and physiological resilience and highlighted the potential of non-pharmacological interventions to restore autonomic balance and reduce vulnerability to stress-related disorders.

Aerobic exercise is well established as a non-pharmacological intervention that improves cardiovascular health. It reduces blood pressure, enhances baroreflex sensitivity, and improves autonomic balance by reducing sympathetic and increasing parasympathetic modulation [15,16]. These adaptations raise the hypothesis that exercise may buffer the adverse cardiovascular effects of chronic ketamine use.

Despite emerging data on ketamine’s acute cardiovascular effects, few studies have systematically evaluated the impact of chronic ketamine exposure on baroreflex sensitivity and heart rate variability—key markers of autonomic function and predictors of cardiovascular risk. The existing literature has largely focused on structural or electrophysiological outcomes, with limited emphasis on dynamic neural regulation. Moreover, the potential of aerobic exercise training to counteract such autonomic impairments in chronic ketamine models remains underexplored. This study addresses this gap by investigating whether aerobic exercise can prevent or reverse ketamine-induced impairments in cardiovascular autonomic regulation, as assessed through baroreflex sensitivity, spectral HRV indices, and pressure variability in conscious animals. By clarifying these interactions, our findings aim to expand the current understanding of non-pharmacological strategies for mitigating ketamine-related cardiotoxicity.

## 2. Materials and Methods

### 2.1. Animals and Experimental Design

Twenty-four male Wistar rats (12 weeks old), with no signs of illness prior to the experiment, were used in this study. Rats were sourced from the institutional animal facility, were specific-pathogen-free, and had no prior experimental manipulation. Inclusion criteria included male Wistar rats aged 12 weeks and in healthy condition; no a priori exclusion criteria were defined. All 24 animals enrolled in the study completed the experimental protocol, and no animals or data points were excluded from the analysis. Each experimental group consisted of six animals (*n* = 6).

Animals were randomly allocated into four groups (*n* = 6 per group) using a simple randomization method based on a random number generator. The allocation was performed by an investigator who was not involved in the subsequent phases of the experiment. The groups were as follows: sedentary control (S), trained control (T), sedentary treated with ketamine (S-Ket), and trained treated with ketamine (T-Ket). To minimize potential confounders, animals were housed in identical polypropylene cages (43 × 23 × 16 cm, three per cage) under controlled temperature (20 ± 2 °C), relative humidity (58%), and a 12 h light/dark cycle (lights on at 6:00 a.m.). Cage locations were rotated weekly, and the order of experimental procedures and measurements was balanced across groups when feasible to reduce systematic bias. Food and water were provided ad libitum.

The sample size was based on previous studies that used similar protocols to detect significant differences in cardiovascular autonomic outcomes in rodents. Although no a priori power analysis was performed, a post hoc power analysis based on the systolic arterial pressure values across the four experimental groups (*n* = 6 per group) was conducted. Using a two-way ANOVA model with two factors (exercise and ketamine), the estimated effect size (f = 1.2) yielded a statistical power greater than 99.9% (α = 0.05; G*Power 3.1). These results confirm that the sample size was adequate to detect meaningful physiological differences.

Investigators were not blinded during the experimental procedures or analysis, as blinding was not feasible in this study due to logistical constraints. However, data were analyzed using coded group identifiers to reduce bias.

**Figure 1:**
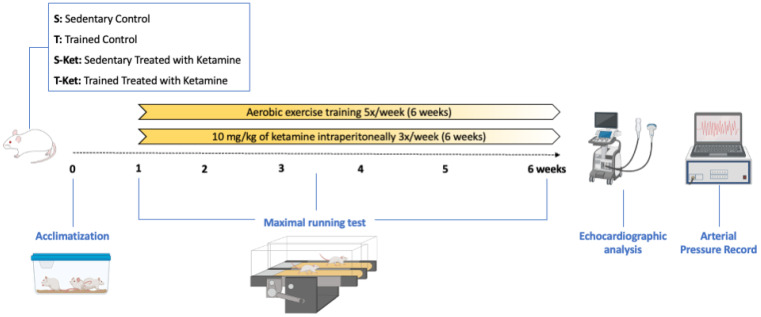
Timeline of experimental design. Rats were allocated to four groups and exposed to treadmill adaptation and an initial maximal effort test (week 1), followed by six weeks of aerobic training and/or ketamine administration. An intermediate test was conducted at week 3 to adjust exercise intensity. In the final week, echocardiographic assessment and surgical catheterization were performed, followed by conscious recordings of arterial pressure, baroreflex sensitivity, and heart rate variability.

A schematic timeline summarizing the experimental procedures—including animal allocation, ketamine administration, exercise training, treadmill testing, echocardiography, catheter implantation, and cardiovascular/autonomic assessments—is presented in Figure 1 to improve clarity.

### 2.2. Ketamine Administration

Ketamine hydrochloride (10 mg/kg) was administered intraperitoneally three times per week for six weeks, using a sterile 1 mL syringe, between 4:00 p.m. and 6:00 p.m., based on the protocol by Jiang et al. [17], to minimize the influence of circadian variation on autonomic and cardiovascular parameters. All groups received injections at the same time of day to ensure consistency and reduce potential confounding by time-dependent physiological fluctuations.

The dose of 10 mg/kg of ketamine administered intraperitoneally three times per week for six weeks was selected based on previously validated protocols in experimental models of chronic exposure [17]. This regimen is considered subanesthetic in rodents and has been widely used to investigate the systemic effects of ketamine, including cardiovascular and autonomic alterations, without inducing deep sedation that would compromise animal locomotion. The estimated translational equivalence using the metabolic correction factor (Km) suggests that this dose in rats corresponds to approximately 1.62 mg/kg in humans, or about 113 mg for a 70 kg adult—a value above the therapeutic dose for depression (0.5 mg/kg IV), but consistent with recreational or abusive exposures observed clinically. Therefore, this approach aims to model repeated and cumulative ketamine exposure, simulating chronic use scenarios with potential cardiotoxicity, as indicated by sustained elevations in blood pressure and impairments in autonomic modulation observed in this and other studies.

### 2.3. Exercise Training Protocol

All animals underwent a maximal treadmill test in the 1st and 6th weeks to assess aerobic capacity and adjust training intensity. Before starting the training protocol, animals were submitted to a 5-day treadmill (AVS, São Paulo, Brazil) adaptation period to habituate them to the equipment and motor noise. During this period, rats ran for 10 min per day at 0.3 km/h. The maximal test began at 0.3 km/h with 3 min stages and 0.3 km/h increments until exhaustion [18,19].

Trained animals performed treadmill running 5 days/week for 6 weeks (60 min/session), at 50–70% of individual maximal speed. An interim test in week 3 adjusted exercise intensity [19].

### 2.4. Echocardiographic Evaluation

Cardiac function was evaluated under light anesthesia (0.5–2% isoflurane in 98% O_2_, 1.5 L/min) using a Vevo 2100 ultrasound system (FujiFilm VisualSonics, Toronto, ON, Canada) and a 13 MHz transducer, following standard guidelines [20].

### 2.5. Hemodynamic and Autonomic Recordings

After anesthesia and catheterization (carotid artery and jugular vein, using Tygon P50), animals recovered for 24 h before data collection. Arterial pressure (AP) and heart rate (HR) were recorded in conscious rats for 30 min via a pressure transducer (Kent Scientific, Torrington, CT, USA) and data acquisition system (WinDaq, 2 kHz) [19].

Throughout the experiment, animals were monitored daily for signs of stress or illness. Isoflurane anesthesia was used to minimize discomfort during surgical procedures. No unexpected adverse events were observed. Humane endpoints were established but not required during the study.

### 2.6. Baroreflex Sensitivity Assessment

Baroreflex sensitivity was evaluated in conscious, freely moving animals 24 h after catheter implantation. Increasing doses of phenylephrine (PE, 2.5–10 µg/kg) and sodium nitroprusside (SNP, 2.5–15 µg/kg) were administered intravenously via the jugular catheter to induce transient pressor and depressor responses, respectively. For each response, the corresponding change in heart rate (HR) was plotted against the change in mean arterial pressure (MAP), and the linear regression slope (ΔHR/ΔMAP, bpm/mmHg) was calculated as the BRS index. At least three responses per agent with linearity >0.9 were considered per animal [19]. All animals were handled prior to testing to reduce stress and were recorded in the morning to minimize circadian variability.

### 2.7. Heart Rate and Blood Pressure Variability

Selected 5 min stable segments were analyzed for pulse interval (PI) and systolic arterial pressure (SAP) variability. Time-domain parameters (SD-PI, rMSSD) and frequency–domain components (VLF-PI, LF-PI, HF-PI) were assessed via Fast Fourier Transform using CardioSeries v2.4 software [19]. For spectral analysis, the following frequency bands were used: very low frequency (VLF-PI: 0.00–0.20 Hz), low frequency (LF-PI: 0.20–0.75 Hz), and high frequency (HF-PI: 0.75–3.00 Hz). These bands were defined according to standard guidelines for spectral analysis in rodents [21].

SAP variability included SD-SAP, Var-SAP, and LF-SAP to estimate sympathetic modulation [21].

### 2.8. Outcomes Measures

The primary outcome measure of this study was arterial pressure, which was used to estimate the required sample size and served as the main parameter for group comparisons. Secondary outcome measures included autonomic, cardiovascular, and echocardiographic parameters. Autonomic modulation was assessed through baroreflex sensitivity, heart rate variability (HRV), and systolic arterial pressure variability (Var-SAP), analyzed in both time and frequency domains using validated indices (e.g., SD-PI, rMSSD, LF/HF power). Additional cardiovascular assessments included heart rate recorded in conscious rats. Cardiac function was evaluated by echocardiographic measurements, including left ventricular volumes, ejection fraction, shortening fraction, and myocardial performance index (MPI). All outcome assessments were performed under standardized and blinded analysis conditions.

### 2.9. Statistical Analysis

Normality of data distribution was assessed using the Shapiro–Wilk test. When assumptions of normality were not met, non-parametric tests were considered, but all variables passed normality criteria. Homogeneity of variances was tested using Levene’s test prior to ANOVA, and all variables met this assumption. Data are expressed as mean ± SEM. Two-way ANOVA tested effects of ketamine and exercise, followed by Tukey’ s post-hoc test when appropriate. The coefficient of determination (R^2^) was used as a measure of effect size, where R^2^ ≤ 0.09 represents a small effect, R^2^ between 0.10 and 0.50 indicates a moderate effect, and R^2^ between 0.51 and 0.99 denotes a large effect [22]. A significance level of α < 0.05 was adopted. All statistical analyses were conducted using GraphPad Prism 9.0 (GraphPad Software, San Diego, CA, USA), with *n* = 6 animals per group.

### 2.10. Use of Artificial Intelligence

Generative artificial intelligence (ChatGPT-4, OpenAI, https://openai.com, accessed on 20 May 2025) was used solely for grammar, structure refinement, and format adaptation of the text. No data, protocols, or analyses were generated by AI tools.

## 3. Results

### 3.1. Body Weight and Aerobic Performance

At baseline, no significant differences were observed in body weight among the experimental groups (S: 395 ± 7 g; T: 394 ± 8 g; S-Ket: 392 ± 8 g; T-Ket: 395 ± 6 g). However, by the end of the protocol, aerobic training led to a significant reduction in body weight in the trained groups (T and T-Ket) compared to the sedentary group (S: 454 ± 7 g; S-Ket: 434 ± 7 g; T: 426 ± 9 g; T-Ket: 422 ± 5 g).

Functional capacity, assessed through a maximal effort treadmill test, was initially similar among groups (S: 13.8 ± 0.8; T: 14.0 ± 0.9; S-Ket: 13.9 ± 1.0; T-Ket: 14.6 ± 1.3 min). After the intervention period, both trained groups exhibited significant improvement in running capacity compared to sedentary groups, with the T-Ket group displaying the highest performance (S: 12.3 ± 1.1; S-Ket: 11.9 ± 0.7; T: 17.0 ± 0.8; T-Ket: 21.0 ± 1.1 min).

### 3.2. Hemodynamic Parameters

**Figure 2:**
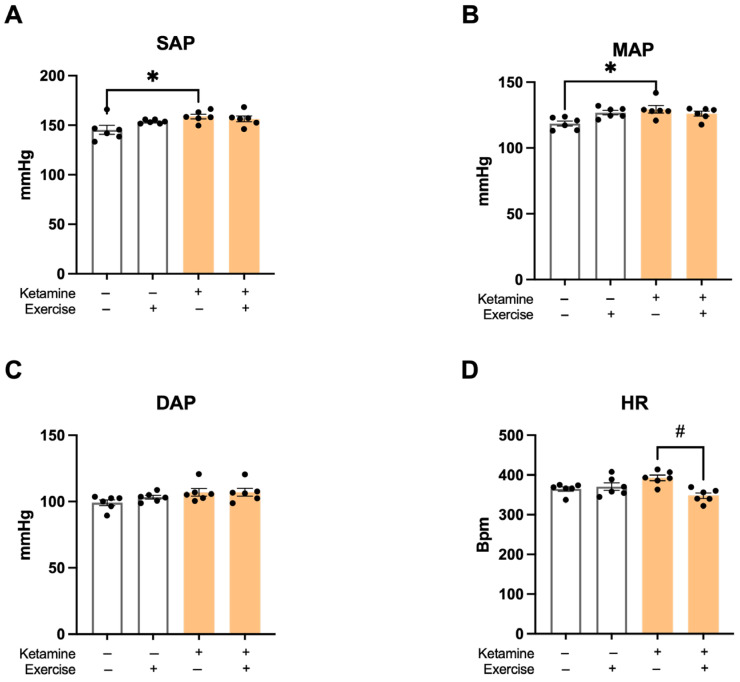
Hemodynamic parameters at the end of the protocol. (**A**) Systolic arterial pressure (SAP), (**B**) mean arterial pressure (MAP), (**C**) diastolic arterial pressure (DAP), and (**D**) heart rate (HR) in sedentary control (S), trained control (T), sedentary with ketamine (S-Ket), and trained with ketamine (T-Ket) groups. Data are expressed as mean ± SEM (*n* = 6 per group). * *p* < 0.05 vs. S; # *p* < 0.05 vs. S-Ket.

The S-Ket group showed significantly elevated SAP compared to the S group (S: 147 ± 2 mmHg; S-Ket: 159 ± 3; T: 153 ± 1; T-Ket: 156 ± 3 mmHg; R^2^ = 0.3575). Mean arterial pressure (MAP) was also higher in the S-Ket group (S: 117 ± 3; S-Ket: 129 ± 3; T: 126 ± 2; T-Ket: 126 ± 2 mmHg; R^2^ = 0.3295), while no significant differences were found in diastolic arterial pressure (DAP) across groups (S: 99 ± 2; S-Ket: 107 ± 4; T: 103 ± 2; T-Ket: 105 ± 1 mmHg) (Figure 2A–C). The T-Ket group presented significantly lower HR values compared to the S-Ket group (S: 381 ± 30; S-Ket: 393 ± 23; T: 361 ± 9; T-Ket: 356 ± 26 bpm; R^2^ = 0.4209) (Figure 2D).

### 3.3. Echocardiographic Findings

**Figure 3:**
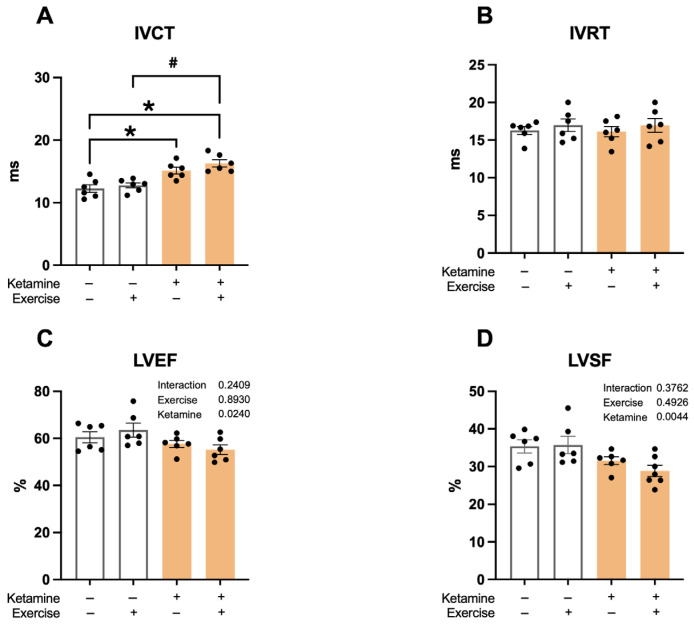
Echocardiographic parameters in sedentary and trained rats, with and without ketamine treatment. (**A**) Isovolumetric contraction time (IVCT), (**B**) Isovolumetric relaxation time (IVRT), (**C**) Left ventricular ejection fraction (LVEF), and (**D**) Left ventricular shortening fraction (LVSF). Data expressed as mean ± SEM (*n* = 6 per group). * *p* < 0.05 vs. S; # *p* < 0.05 vs. S-Ket.

Ketamine exposure resulted in prolonged isovolumetric contraction time (IVCT—S: 12.3 ± 0.6; S-Ket: 15.1 ± 0.8; T: 12.8 ± 0.6; T-Ket: 16.3 ± 0.7 ms; R^2^ = 0.6306) (Figure 3A), as well as reduced aortic ejection time (R^2^ = 0.2092), left ventricular ejection fraction (LVEF—S: 62 ± 2; S-Ket: 58 ± 2; T: 64 ± 3; T-Ket: 56 ± 2%; R^2^ = 0.2174) (Figure 3C), and shortening fraction (LVSF—S: 35 ± 2; S-Ket: 28 ± 1; T: 33 ± 2; T-Ket: 36 ± 2%; R^2^ = 0.3944) (Figure 3D). Aerobic exercise modulated left ventricular systolic (R^2^ = 0.4272) and diastolic (R^2^ = 0.2729) volumes. Additionally, ketamine significantly worsened the myocardial performance index (R^2^ = 0.2009). No differences were observed in the left atrial-to-aortic ratio among the studied groups (Table 1).

### 3.4. Baroreflex Sensitivity

**Figure 4:**
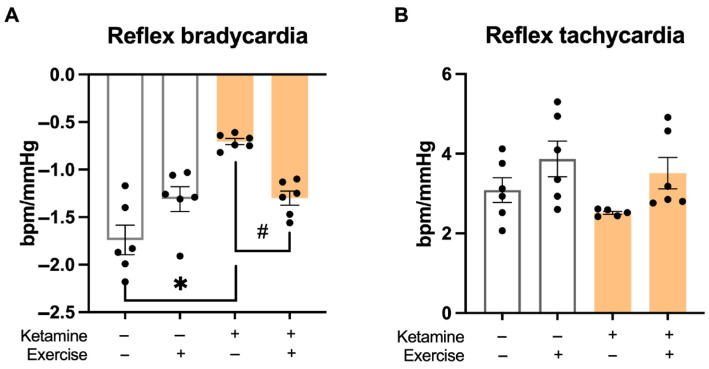
Baroreflex sensitivity in response to pharmacological stimuli. (**A**) Bradycardic response to phenylephrine-induced hypertension and (**B**) tachycardic response to sodium nitroprusside-induced hypotension. Data are presented as mean ± SEM (*n* = 6 per group). * *p* < 0.05 vs. S; # *p* < 0.05 vs. S-Ket.

Baroreflex sensitivity to bradycardic responses was significantly reduced in the S-Ket group compared to all other groups (Figure 4A). Sensitivity to tachycardic responses was also lower in the S-Ket group compared to the T group (Figure 4B), indicating autonomic impairment associated with ketamine exposure.

### 3.5. Heart Rate Variability (HRV)

In the time-domain analysis, there were no differences in the SD-PI or variance (Var-PI) of the pulse interval. However, the root mean square of successive differences (rMSSD) was significantly reduced in the S-Ket group, suggesting diminished parasympathetic modulation (Table 2).

**Figure 5:**
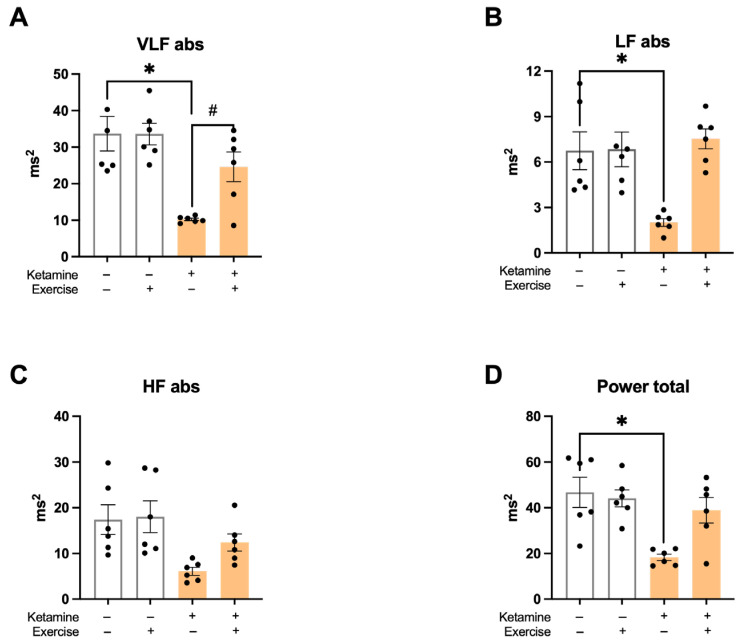
Heart rate variability in the frequency domain. (**A**) Very low-frequency band (VLF-PI abs), (**B**) low-frequency band (LF-PI abs), (**C**) high-frequency band (HF-PI abs), and (**D**) total power in pulse interval analysis. Data expressed as mean ± SEM (*n* = 6 per group). * *p* < 0.05 vs. S; # *p* < 0.05 vs. S-Ket.

In the frequency–domain analysis, ketamine significantly reduced HRV. The S-Ket group exhibited lower values in the very low-frequency (VLF-PI) and low-frequency (LF-PI) bands compared to all other groups (Figure 5A,B). High-frequency (HF-PI) components were also decreased by ketamine (S: 16.1 ± 3.0; S-Ket: 6.1 ± 1.3; T: 20 ± 5; T-Ket: 12 ± 3 ms^2^). Total power was significantly lower in the S-Ket group compared to the S group (S: 45.6 ± 6.3; S-Ket: 18.3 ± 2.1; T:44 ± 6; T-Ket: 38 ± 8 ms^2^) (Figure 5C,D), indicating overall autonomic impairment.

### 3.6. Systolic Arterial Pressure Variability (SAPV)

No significant differences were observed across groups in the SD-SAP or LF-SAP bands. However, variance in systolic arterial pressure (Var-SAP) was significantly higher in the S-Ket group compared to the S and T-Ket groups (Table 2), suggesting an altered autonomic regulation of vascular tone induced by ketamine, partially restored by exercise.

## 4. Discussion

This study examined the chronic effects of ketamine hydrochloride on hemodynamic parameters, autonomic modulation, and baroreflex sensitivity and demonstrated that aerobic exercise can counteract several of these negative outcomes. Over a six-week period, ketamine administration elevated systolic and mean arterial pressure while reducing baroreflex sensitivity—especially the bradycardic response. Moreover, a generalized reduction in autonomic modulation was evident in spectral analysis, suggesting early-stage cardiovascular autonomic dysfunction.

While these findings support a pathophysiological role for oxidative imbalance, an important limitation of the present study is the absence of direct biochemical assessments of oxidative stress or inflammatory markers. Although our functional and hemodynamic results support the hypothesis that oxidative stress may underlie the cardiovascular effects of chronic ketamine exposure, no tissue or plasma measurements of markers such as malondialdehyde (MDA), superoxide dismutase (SOD), or tumor necrosis factor-alpha (TNF-α) were performed. This limits our ability to directly confirm oxidative or inflammatory mechanisms. Nonetheless, existing evidence lends support to this hypothesis. For instance, Cetin et al. [23] demonstrated that ketamine increases oxidative stress in cardiac tissue—marked by elevated MDA and decreased antioxidant enzymes—and that disulfiram attenuates this damage in a dose-dependent manner. Incorporating such biomarkers in future studies will be essential to clarify the mechanistic pathways through which ketamine induces cardiovascular dysfunction and to confirm the protective effects of exercise at the molecular level.

Ketamine, a non-competitive NMDA receptor antagonist, has a growing range of clinical applications beyond anesthesia, notably in psychiatry and pain management. Despite its therapeutic value, ketamine exerts marked cardiovascular effects, including elevated blood pressure and heart rate due to sympathetic activation. Most of the existing literature focuses on acute administration, where transient hypertensive effects are documented (e.g., increases of ~13 mmHg) that normalize within hours [10,11]. However, our results challenge this transience by demonstrating that chronic administration (10 mg/kg for 6 weeks) leads to sustained elevations in both systolic (~11 mmHg) and mean (~12 mmHg) arterial pressures.

Interestingly, our data showed that mean arterial pressure (MAP) increased significantly in ketamine-treated animals, despite no statistical difference in diastolic arterial pressure (DAP). Since MAP is a time-weighted average more influenced by DAP than by systolic arterial pressure (SAP), this finding initially seems counterintuitive. However, the concurrent increase in SAP—without a compensatory reduction in DAP—was sufficient to elevate MAP. This may reflect ketamine’s sympathomimetic effects, which include enhanced cardiac output and increased aortic stiffness via β2-adrenoceptor activation. Given that SAP is predominantly determined by central elastic arteries (e.g., aorta), while DAP reflects resistance in smaller downstream vessels, our findings suggest that the vascular impact of chronic ketamine exposure may primarily affect conduit arteries. This interpretation is supported by clinical data from Ansari et al. [24], who reported transient increases in SBP and DBP during ketamine infusion, with a more pronounced rise in systolic pressure during early treatment phases. These hemodynamic changes may also be explained by the direct vascular actions of ketamine. Bevan et al. [25] demonstrated that ketamine binds directly to both α1- and β2-adrenoceptors, with higher affinity for β2 sites. Such interactions may induce vasoconstriction or vasodilation depending on the vascular bed and receptor distribution, contributing to the observed increase in systolic pressure and overall MAP without significant change in DAP.

These sustained hypertensive effects appear to result from impaired neural regulation of arterial pressure. Specifically, ketamine-exposed animals exhibited blunted baroreflex responses to both hypertensive (bradycardic) and hypotensive (tachycardic) stimuli, suggesting compromised autonomic function. This interpretation is further supported by the observed reductions in heart rate variability (HRV), including diminished rMSSD and spectral power in both the low- and high-frequency bands—indices that reflect parasympathetic modulation. Together, these findings point to a disruption in sympathovagal balance caused by chronic ketamine exposure.

Baroreflex sensitivity (BRS), a reliable indicator of autonomic function, is known to decline in conditions of heightened sympathetic activity and improve with parasympathetic predominance [26]. Our findings align with studies indicating reduced BRS in both clinical and experimental models of cardiovascular dysfunction [27,28,29,30], reinforcing the view that prolonged ketamine exposure may precipitate or exacerbate cardiovascular dysautonomia.

Additionally, our study showed that ketamine reduced heart rate variability (HRV) and systolic arterial pressure variability (SAPV)—both key indicators of autonomic balance. These changes were evident in time-domain measures (e.g., reduced rMSSD) and across all spectral bands (VLF, LF, HF), indicating a significant loss of parasympathetic tone and overall autonomic control.

From a physiological standpoint, each HRV spectral band reflects distinct components of autonomic regulation. The VLF band (0.00–0.20 Hz) is often associated with thermoregulatory and hormonal influences. The LF band (0.20–0.75 Hz) reflects both sympathetic and parasympathetic activity, while the HF band (0.75–3.00 Hz) is primarily mediated by vagal tone and closely linked to respiratory sinus arrhythmia [21]. Thus, the reductions observed in LF and HF power support the hypothesis that chronic ketamine exposure disrupts sympathovagal balance, mainly through attenuation of parasympathetic modulation.

These results are consistent with previous animal studies showing that chronic ketamine administration impairs cardiac function, increases arrhythmogenic risk, and alters autonomic responsiveness [13]. Interestingly, β-blockers have been shown to partially reverse these effects, underscoring the role of sympathetic overactivity [13].

Notably, only the group that combined aerobic training with ketamine exposure (T-Ket) showed a significant reduction in resting heart rate, while neither ketamine nor exercise alone produced this effect. This finding may reflect a compensatory interaction between interventions with opposing autonomic influences. While chronic ketamine use is associated with sympathetic overactivity and impaired baroreflex function [31], aerobic exercise is well known to increase vagal tone and reduce resting sympathetic drive [32,33]. Therefore, the heart rate reduction observed in the T-Ket group may result from a partial restoration of parasympathetic dominance, mediated by exercise, that offsets the sympathoexcitatory effects of ketamine. This interpretation is supported by our HRV data, which showed higher rMSSD and HF power in trained animals exposed to ketamine, suggesting that regular aerobic exercise can modulate autonomic imbalance and restore resting cardiac control even in the presence of pharmacological stress.

The novelty of our study lies in demonstrating that aerobic exercise training restores cardiovascular autonomic function and baroreflex sensitivity in animals chronically exposed to ketamine. Exercise attenuated the ketamine-induced increase in blood pressure, normalized baroreflex responses, and partially restored HRV and SAPV parameters. These findings contribute to the growing body of evidence supporting physical training as a non-pharmacological intervention capable of enhancing autonomic regulation.

Beyond cardiovascular outcomes, trained animals exhibited reduced body weight compared to sedentary controls, despite ketamine not exacerbating weight gain. Although our study did not focus on metabolic outcomes, this observation aligns with conflicting findings in the literature. While some studies show ketamine increasing food intake in anorexia models [34], our results suggest that chronic administration does not promote significant weight gain, particularly in the presence of exercise.

Interestingly, exercise enhanced the functional capacity of all trained animals, as measured by treadmill performance. Notably, animals in the ketamine + training group (T-ket) outperformed the training-only group. This unexpected result may relate to synergistic effects on neurotransmitter systems, including serotonergic and dopaminergic modulation, known to be influenced by both exercise and ketamine [35,36]. These mechanisms may also contribute to reduced central fatigue and enhanced endurance [37].

Previous studies have consistently shown that physical training improves cardiovascular autonomic regulation. While heart rate and blood pressure rise acutely during exercise, repeated sessions lead to improved vagal tone and reduced sympathetic overactivity at rest [38,39]. Moreover, some studies demonstrate that removing baroreceptor feedback negates the cardiovascular benefits of training in hypertensive rats, highlighting the essential role of baroreceptors [40]. Other research suggests that exercise may downregulate β2-adrenergic receptor sensitivity, provide vascular protection, and reduce hypertension risk [41,42].

A particularly relevant finding in our study was that exercise normalized SAPV, reversing the sympathetic overactivity induced by ketamine. Given ketamine’s known sympathomimetic actions via α-1 and β-2 adrenoceptors [25,43], this reversal supports the hypothesis that exercise exerts protective effects through autonomic modulation.

Taken together, our results support the hypothesis that aerobic exercise training prevents impairments in cardiovascular autonomic modulation caused by chronic ketamine exposure. This is in line with previous findings demonstrating the beneficial effects of physical exercise on heart rate variability, baroreflex sensitivity, and inflammatory modulation in models of cardiovascular dysfunction. These findings reinforce the concept that regular aerobic exercise can counteract sympathovagal imbalance and preserve autonomic regulation even under pharmacological stress.

Moreover, our data are consistent with recent evidence linking reduced parasympathetic activity to both physiological and affective dysregulation. For instance, Gullett et al. [14] systematically reviewed studies showing that lower HRV—particularly in the high-frequency band and RMSSD—is associated with negative affect and greater vulnerability to stress in non-clinical populations. This further supports the idea that restoring autonomic balance through aerobic training may serve not only as a cardiovascular protective strategy but also to enhance broader emotional and stress resilience.

Although these findings are based on a rodent model, they suggest that aerobic exercise may provide protective cardiovascular autonomic effects against the detrimental impact of chronic ketamine exposure. Given the conserved nature of autonomic regulatory mechanisms across mammals, our results may hold relevance to human physiology, particularly in contexts of chronic ketamine use, such as psychiatric treatment or substance misuse. Nevertheless, translational application requires caution due to interspecies differences in metabolism, neurophysiology, and drug sensitivity.

Some limitations of this study should be acknowledged. First, the relatively small sample size (*n* = 6 per group), although consistent with previous studies, may reduce the statistical power to detect subtle effects. Second, the study was conducted exclusively in male Wistar rats, which limits the generalizability of the findings. Although this choice aimed to minimize variability in cardiovascular and autonomic parameters, it overlooks important sex differences. There is growing evidence that females may exhibit different susceptibilities to ketamine’s neurochemical and cardiovascular effects—potentially due to hormonal modulation and differential receptor expression—which may influence both autonomic regulation and pharmacodynamic responses [44]. Future studies should therefore include female animals to assess sex-specific effects and enhance the translational value of preclinical models.

Third, although the dosing regimen was designed to simulate repeated ketamine exposure, extrapolation to human physiology must be approached with caution. Rodents differ from humans in metabolic rate, baseline autonomic tone, and receptor distribution, all of which may influence cardiovascular outcomes and the modulatory effects of interventions such as aerobic exercise. Addressing these interspecies differences in future translational studies, including complementary pharmacokinetic assessments, will be essential.

Finally, investigators were not blinded during experimental procedures or data collection, which may have introduced observer bias. To mitigate this risk, several strategies were implemented: data analysis was performed using coded group identifiers, all procedures followed standardized protocols, and data collection was randomized when feasible. Nonetheless, we recognize that the absence of full blinding remains a limitation, as highlighted in the preclinical reproducibility literature [45]. Future studies should aim to incorporate blinding and assess inter-rater reliability to further enhance methodological rigor.

## 5. Conclusions

This study demonstrated that chronic administration of ketamine hydrochloride leads to sustained increases in arterial pressure, impaired baroreflex sensitivity, and reduced cardiovascular autonomic modulation in Wistar rats. Importantly, aerobic exercise training effectively mitigated these adverse effects, restoring baroreflex function and improving heart rate and blood pressure variability.

These findings suggest that regular aerobic exercise may serve as a protective strategy against ketamine-induced cardiovascular dysregulation. Given ketamine’s expanding clinical use and known sympathomimetic properties, the incorporation of physical training into therapeutic protocols could represent a safe and accessible non-pharmacological intervention to preserve autonomic cardiovascular function.

While promising, these results were obtained in an experimental animal model and must be interpreted with caution when considering translational applications. Further research—including studies with varied doses, treatment durations, and eventually human trials—is necessary to confirm the efficacy of exercise in counteracting the cardiovascular effects of ketamine and to guide clinical recommendations for integrated, lifestyle-based treatment approaches.

## Figures and Tables

**Table 1:** Echocardiographic parameters in sedentary and trained rats, with and without ketamine treatment.

Groups Measurements	S	S-Ket	T	T-Ket		
					*p* value ANOVA	*p* value GLM
Doppler Flux						
AET (ms)	64 ± 1.0	61 ± 1.4	64 ± 0.9	62 ± 1.4	0.2274	<0.05 ^#^
MPI	0.47 ± 0.0	0.57 ± 0.0	0.48 ± 0.0	0.55 ± 0.0	0.2243	<0.05 ^#^
Morphometric Measurements						
LV Vol;d (µL)	296 ± 16	276 ± 12	231 ± 11	256 ± 26	0.1345	<0.05 *
LV Vol;s (µL)	118 ± 8	117 ± 10	82 ± 11	95 ± 10	0.0576	<0.05 *
LA/Ao	0.7 ± 0.0	0.6 ± 0.0	0.7 ± 0.0	0.6 ± 0.0	0.2144	<0.05 ^#^

Values expressed as mean ± SEM. AET: aortic ejection time; MPI: myocardial performance index; LV Vol;s: systolic volume; LV Vol;d: diastolic volume; LA/Ao: left atrial-to-aortic ratio. Data are expressed as mean ± SEM (*n* = 6 per group). * *p* < 0.05 vs. S; ^#^ *p* < 0.05 vs. S-Ket.

**Table 2:** Cardiovascular autonomic modulation indices: time-domain HRV and SAP variability.

Groups Measurements	S	S-Ket	T	T-Ket		
					*p* value ANOVA	*p* value GLM
HRV (time domain)						
SD-PI (ms)	8.6 ± 0.7	7.1 ± 0.6	8.5 ± 0.8	10.3 ± 1.1	0.14	-
Var-PI (ms^2^)	69.8 ± 13	45.9 ± 7	76.7 ± 14	79.9 ± 27	0.60	-
rMSSD (ms)	7.2 ± 0.6	4.8 ± 0.5 ^#^	7.1 ± 0.7	6.3 ± 0.7 ^#^	0.07	<0.05
SAPV (time and frequency domain)						
SD-SAP (mmHg)	5.3 ± 0.5	6.2 ± 0.7	5.9 ± 0.5	4.7 ± 0.2	0.20	-
Var-SAP (mmHg^2^)	25.4 ± 1.8	46.3 ± 9.2 *	29.1 ± 6.2 *	23.3 ± 1.3 *	0.00	<0.05
LF-SAP (mmHg^2^)	5.9 ± 0.7	6.1 ± 1.1	7.0 ± 1.3	5.4 ± 0.4	0.68	-

HRV: heart rate variability; rMSSD: root mean square of successive differences; SD-PI: standard deviation of pulse interval; Var-PI: variance of pulse interval; SD-SAP: standard deviation of systolic arterial pressure; Var-SAP: variance of systolic arterial pressure; LF-SAP: low-frequency band of SAP. Values presented as mean ± SEM (*n* = 6 per group). Statistical analysis was primarily performed using two-way ANOVA to evaluate the main effects of ketamine, exercise, and their interaction. When relevant, General Linear Model (GLM) results are also reported for confirmation or exploratory purposes. * *p* < 0.05 vs. S; ^#^ *p* < 0.05 vs. S-Ket.

## Data Availability

The raw data supporting the findings of this study, including HRV spectra and echocardiographic traces, are available on Zenodo: https://doi.org/10.5281/zenodo.16748436, accessed on 20 May 2025.

## Abbreviations

The following abbreviations are used in this manuscript:

AET	Aortic ejection time
BRS	Baroreflex sensitivity
DAP	Diastolic arterial pressure
HF	High frequency band
HR	Heart rate
HRV	Heart rate variability
IVCT	Isovolumetric contraction time
LF	Low frequency band
LV Vol;d	Left ventricular diastolic volume
LV Vol;s	Left ventricular systolic volume
LVEF	Left ventricular ejection fraction
LVSF	Left ventricular shortening fraction
MAP	Mean arterial pressure
MPI	Myocardial performance index
PI	Pulse interval
rMMSD	Root mean square of successive differences
S	Sedentary control
S-Ket	Sedentary with ketamine
SAP	Systolic arterial pressure
SD-PI	Standard deviation of pulse interval
SEM	Standard error of mean
T	Trained control
T-Ket	Trained with ketamine
Var-PI	Variance of pulse interval
VLF	Very low frequency band
